# Supplementary material for: Electrically-driven single-photon sources based on colloidal quantum dots with near-optimal antibunching at room temperature
Source: Nat Commun. 2017 Oct 26;8:1132. doi: 10.1038/s41467-017-01379-6 (PMC5656660; doi:10.1038/s41467-017-01379-6)
Supplement: Supplementary file 1 — Supplementary Information [file 41467_2017_1379_MOESM1_ESM.pdf]

Supplementary Figures

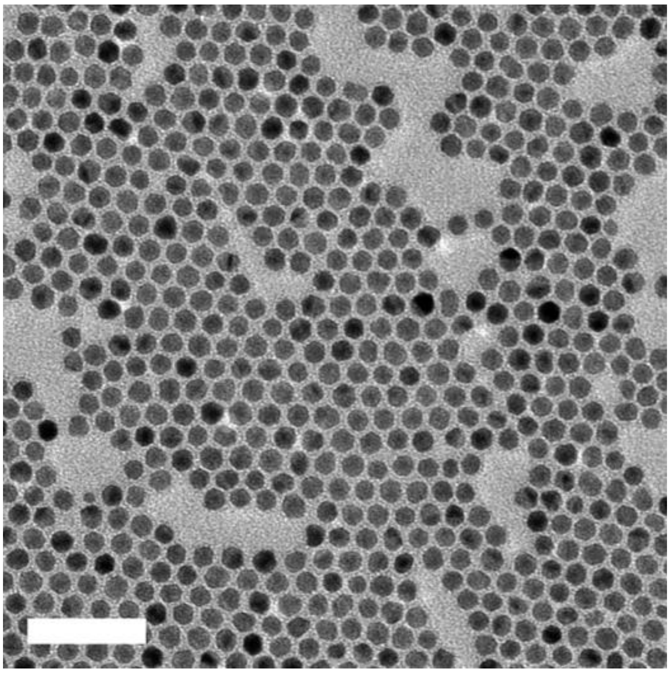

Supplementary Figure 1. TEM image of quantum dot sample. Typical diameters of CdSe/CdS core/shell quantum dots are around 10 nm. Scale bar, 50 nm.

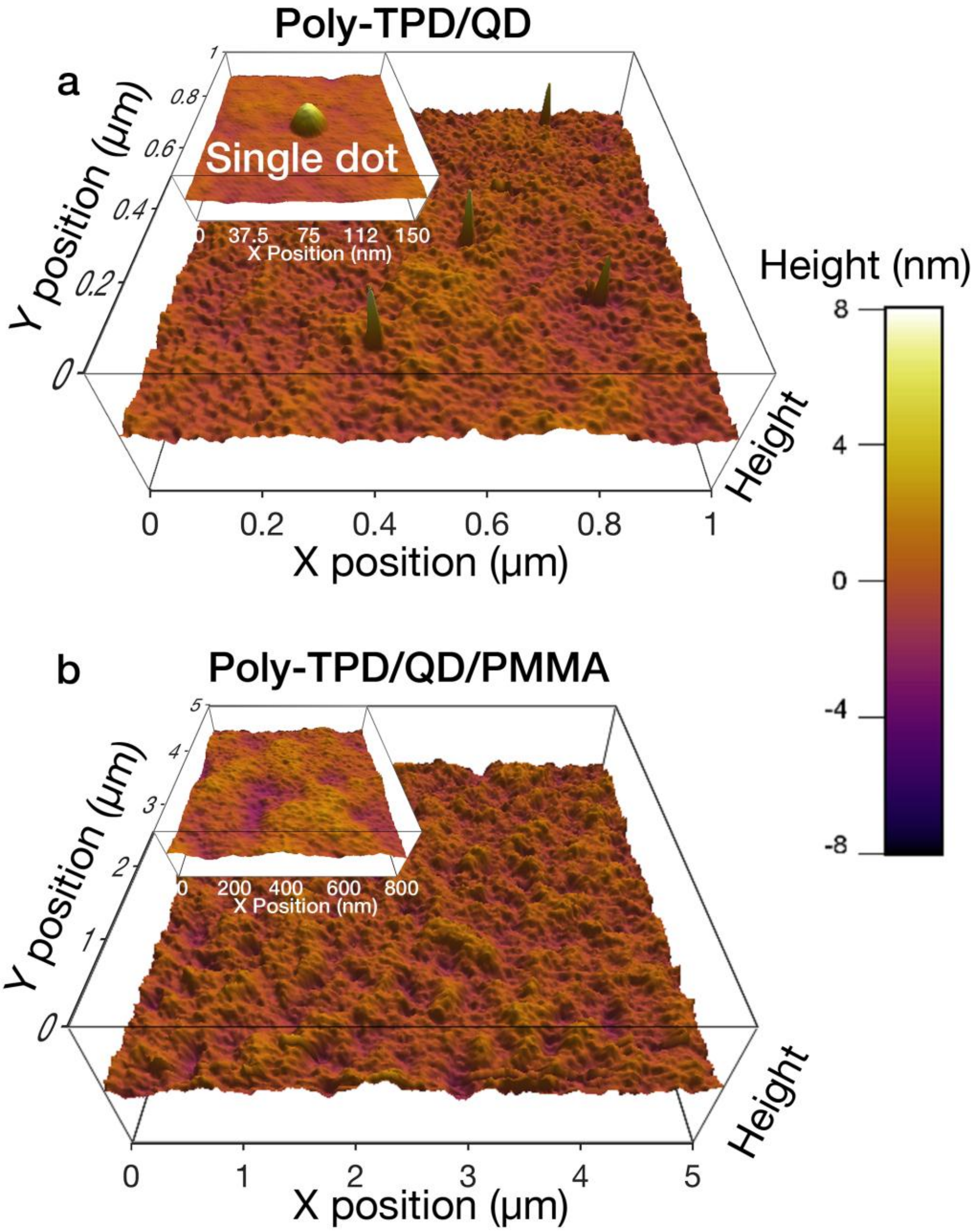

Supplementary Figure 2. Atomic force microscope images of samples. a, A typical Atomic force microscope (AFM) image of isolated quantum dots on Poly-TPD (1um×1um). Inset: Zoom-in scan of a 150 nm×150 nm area with one dot in this region. **b,** A typical AFM image of the same sample after depositing a 12-nm thick PMMA layer (5um×5um). Inset: Zoom-in scan of a 0.8 um×0.8 um area from this region. No dots can be found in multiple measurements, indicating that all the isolated dots were buried in the 12-nm PMMA layer.

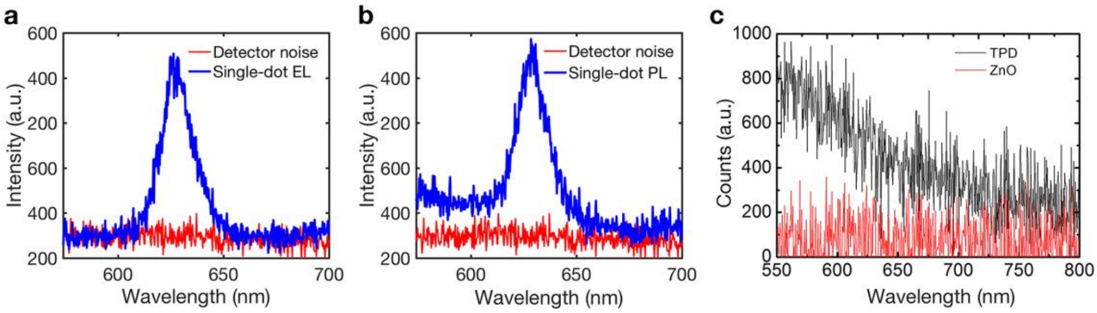

Supplementary Figure 3. Origin of photoluminescence background emission. a, Electroluminescence (EL, driven at 2.8 V) and **b,** photoluminescence (PL, excitation: 450nm c.w. laser) spectra from the same bright spot. All the experimental settings for detectors are same. Note that the PL spectrum contained background emission from Poly-TPD. **c,** Photoluminescence spectra of Poly-TPD (black curve) and ZnO (red curve) under same optical excitation conditions as **b.** Within the spectral range under consideration (600-650 nm), ZnO shows negligible emission comparing to that from Poly-TPD.

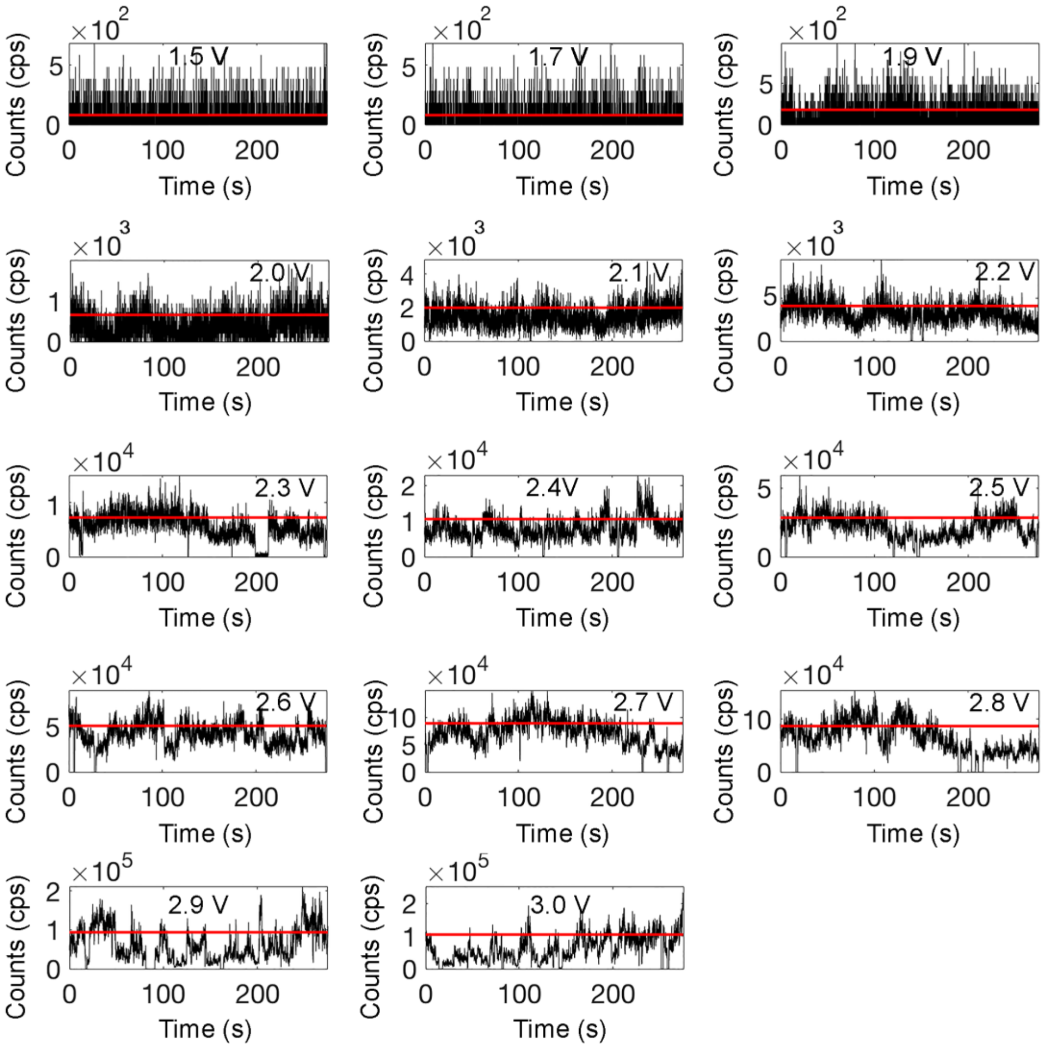

Supplementary Figure 4. Time trace of EL intensity at different driven voltages. Red lines show the extracted average counts, which were plotted in Fig. 1f. It should be pointed out that, due to drifting of the sample holder, the extracted values provided low bounds for the emitting rates of the single-dot EL. Time bin was chosen as 40 ms.

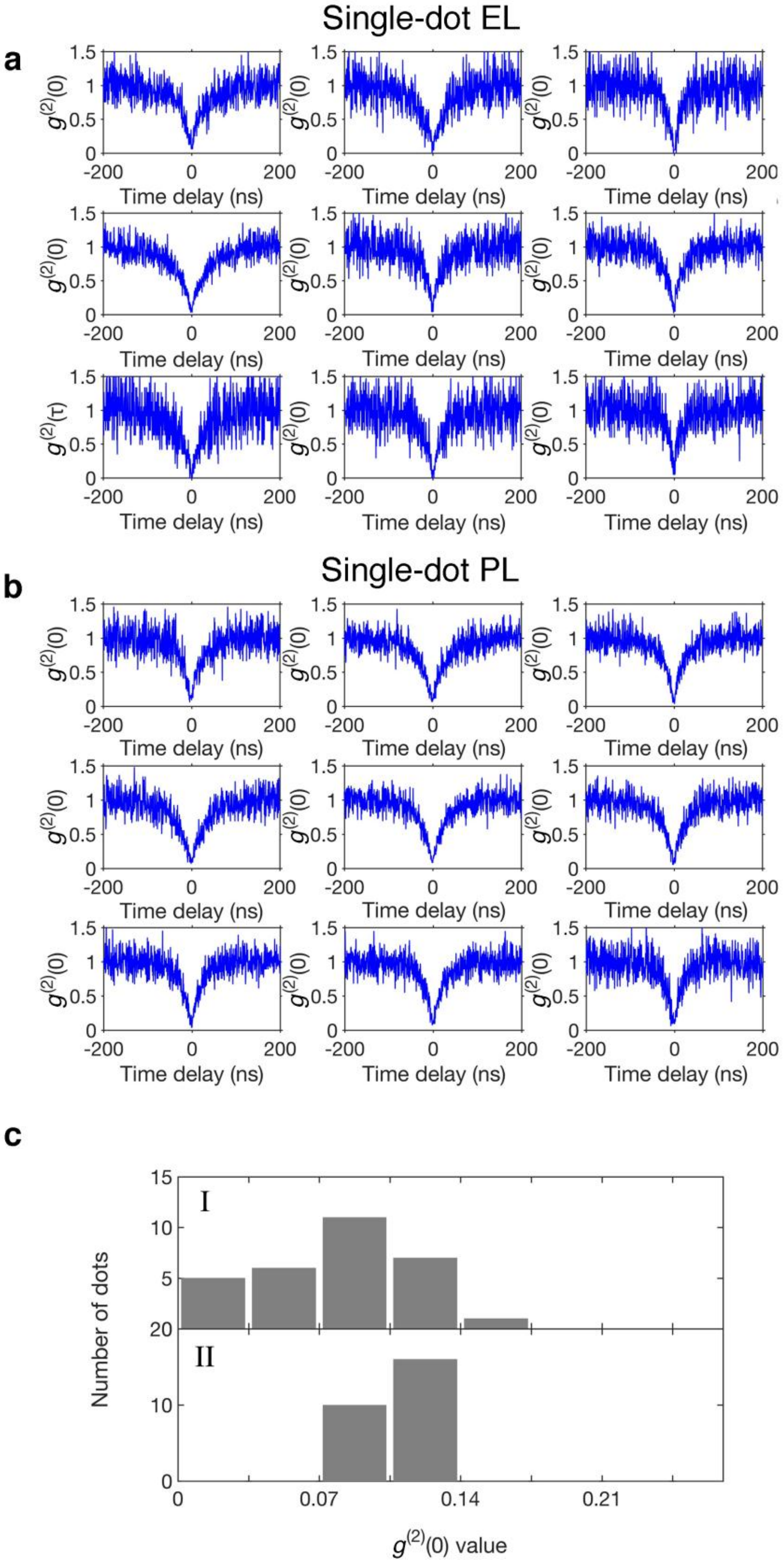

Supplementary Figure 5. Statistics of $g^{(2)}$(0) distributions. Representative EL (**a**) and PL (**b**) $g^2(t)$ curves and $g^{(2)}(0)$ distributions measured using samples from different batches. EL $g^{(2)}(0)$ are determined as 0.08, 0.08, 0.01, 0.07, 0.02, 0.07, 0.6, 0.03 and 0.07 respectively. PL $g^{(2)}(0)$ are determined as 0.14, 0.10, 0.07, 0.13, 0.12,

0.11, 0.12, 0.14 and 0.14 respectively. **c,** Histograms of the $g^{(2)}(0)$ values measured from 30 isolated electroluminescence spots in the devices with approximately 36% of the data points below 0.07 (I), and 26 isolated photoluminescence spots on bare quartz substrates with all of the data points higher than 0.07 (II). Regarding the statistical results for the $g^{(2)}(0)$ of all electro-excited single dots (0.08±0.04), the standard deviation is relatively large. One of the major reasons for such spread distribution of $g^{(2)}(0)$ is due to fluctuation of the thicknesses of PMMA layer, which modulates electron injection rates and thus affects charge balance. At optimal conditions, the EL intensity from single quantum dot shows non-blinking-like time trace at low applied voltages, as shown in Supplementary Fig. 4. In such cases, the $g^{(2)}(0)$ values are generally smaller than the lower limit of $g^{(2)}(0)$ value (~0.07) from photo-excited quantum dots. We would like to note that the uncertainty of the $g^{(2)}(0)$ values has been taken into consideration in such comparison (e.g., the $g^{(2)}(0)$ value shown in Fig. 1e is 0.045 ± 0.005 without any background subtraction). At non-optimal conditions, the unbalanced charge injection results in long-lived charged states in the quantum dots which reduce the average intensity. As a consequence, the signal to noise (mainly from dark counts of APD) ratio is diminished. Considering that the coincident counts at $\tau = 0$ are almost zero, the diminished signal to noise ratio degrades the $g^{(2)}(0)$ values, which brings wide distributions of the statistical results.

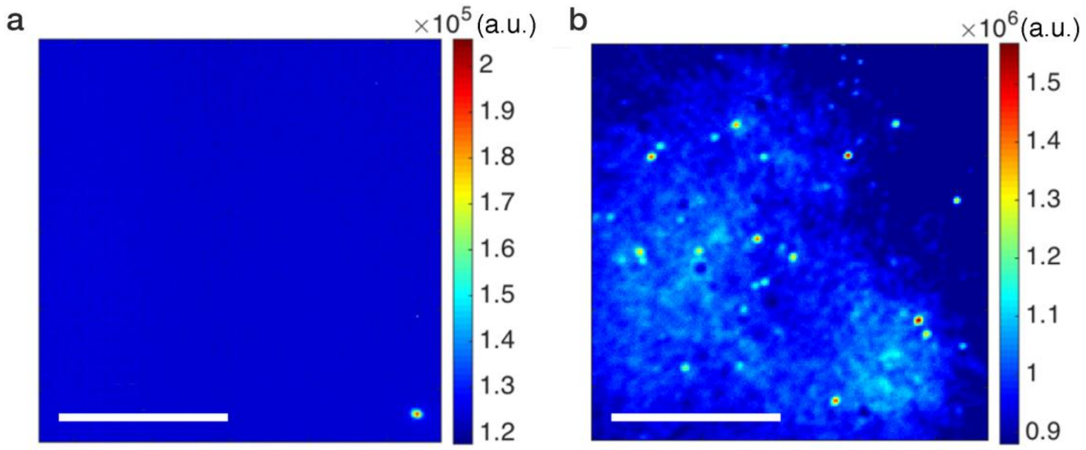

Supplementary Figure 6. EL and PL images of a device without the PMMA layer. **a,** EL image shows only one bright spot while **b,** the PL image (from the same region) shows a dense distribution of isolated quantum dots in the same field of view. Scale bar, 10 nm.

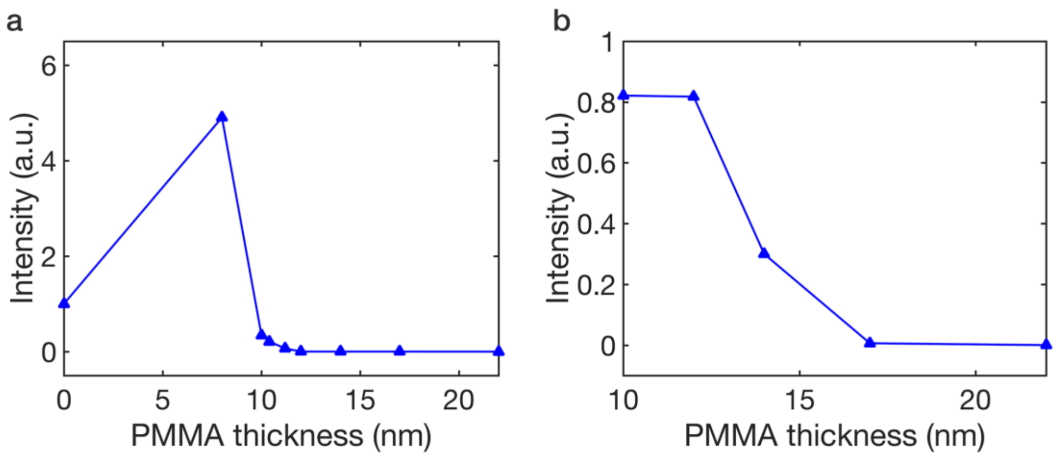

Supplementary Figure 7. The effect of PMMA film thickness. a, The dependence of Poly-TPD intensity (integrated between 400 and 500 nm) on the thickness of the PMMA layer. When the device was driven at a relatively high voltage of 2.8 V, emission from Poly-TPD was negligible and only the emission from quantum dots was observed when the average thickness of the PMMA film reached 12 nm. **b,** The dependence of EL intensity of quantum dots (integration between 600 and 660 nm) on the thickness of the PMMA layer. When the devices were driven at 2.8 V, emission from the quantum dots vanished when the average thickness of PMMA film exceeded ~17 nm.

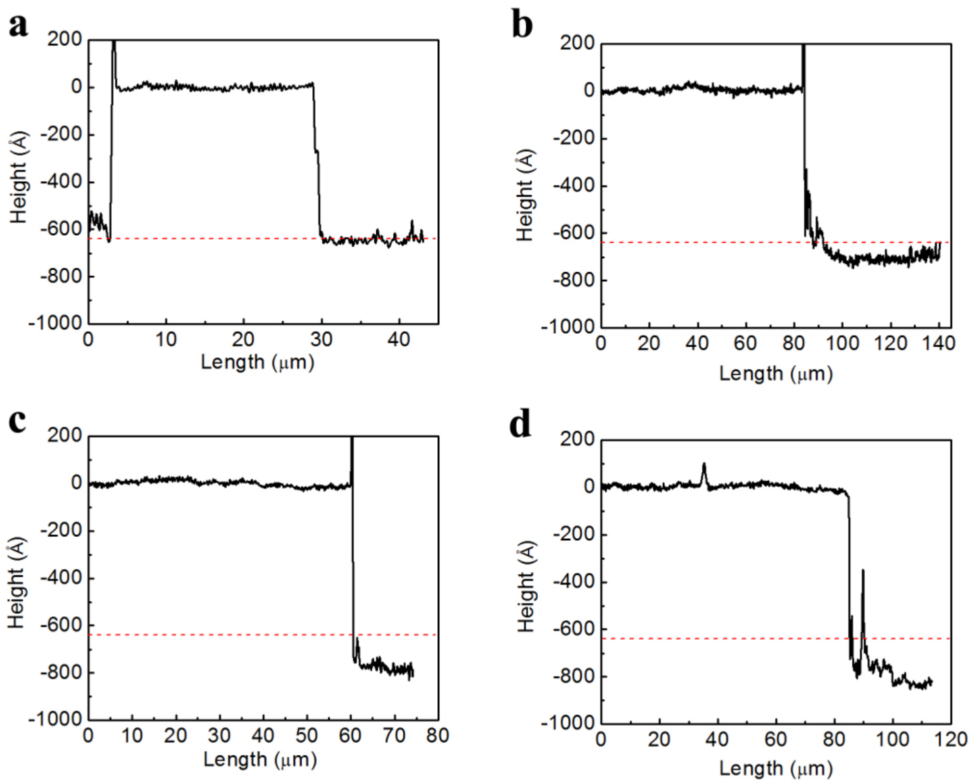

Supplementary Figure 8. PMMA film thickness measurement. Step profiler measurements of samples with different PMMA concentrations in acetone as 0 mg mL^{-1} (**a**), 0.75 mg mL^{-1} (**b**), 1.0 mg mL^{-1} (**c**) and 1.5 mg mL^{-1} (**d**). And the corresponding thicknesses are measured as 0 nm, 8 nm, 12 nm and 17 nm, respectively. The red dashed lines are shown as visual guides.

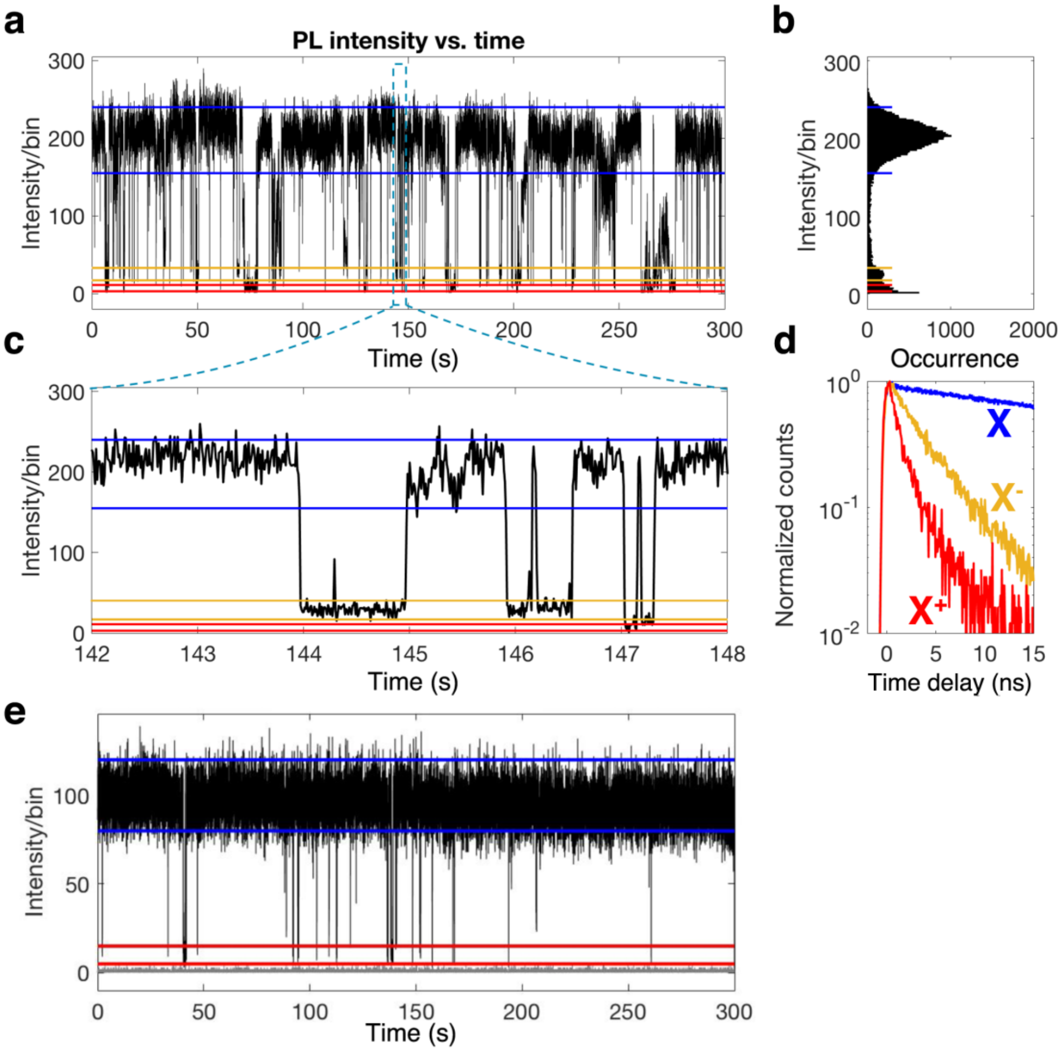

Supplementary Figure 9. Characterization of the trion states. a, Time trace of PL intensity of a single quantum dot under a relatively high pumping power (N_{av}=0.6). **b,** Histogram of intensity distribution from **a** indicates two dim states. **c,** Magnified time trace curve shows two distinct dim states, which should correspond to two trion states. **d,** Lifetime measurements of exciton and two trion states based on the data extracted from the bright state (defined by two blue lines), X^- state (defined by two orange lines) and X^+ state (defined by two red lines) shown in **b**, respectively. Note that we used a high-optical-power (N_{av}=0.6) excitation to generate sufficient trion states. The dots used in our experiments exhibit non-blinking PL properties under low optical power excitation (N_{av}=0.3), as shown in **e**. Time bin was chosen as 10 ms in the measurements.

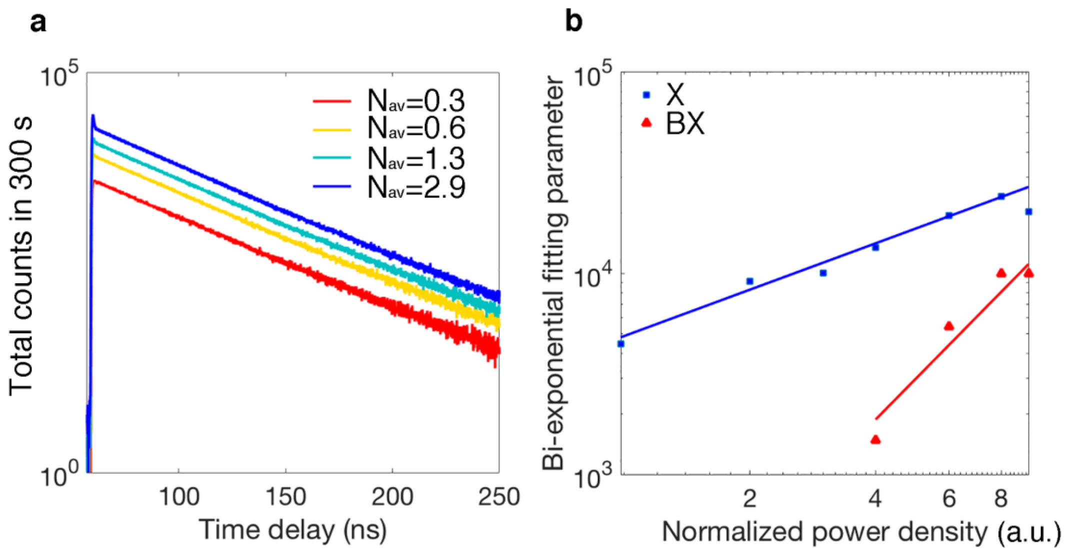

Supplementary Figure 10. Measurement of bi-exciton decay. a, Fluorescence decay curves at different optical excitation power. As the excitation power increased, the emerging sharp peaks indicated the occurrence of bi-exciton emission. **b,** The extracted single exciton and bi-exciton components as functions of excitation power that give expected power dependences (solid fitting lines) for both excitons and bi-excitons.

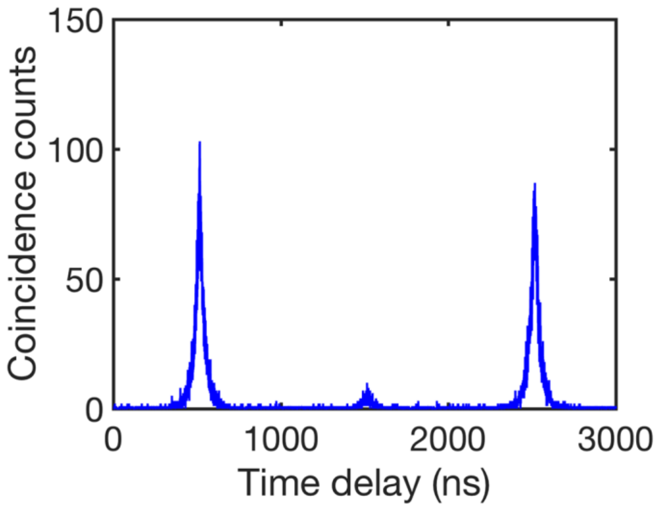

Supplementary Figure 11. Pulsed Photoluminescence $g^{(2)}(\tau)$ measurement. According to Supplementary Ref. 1, the area ratio between centre peak and side peak is calculated as 0.078, which equals to the ratio of fluorescence QY of the bi-excitons to excitons. The experiment is done under low optical power excitation (N_{av}=0.02).

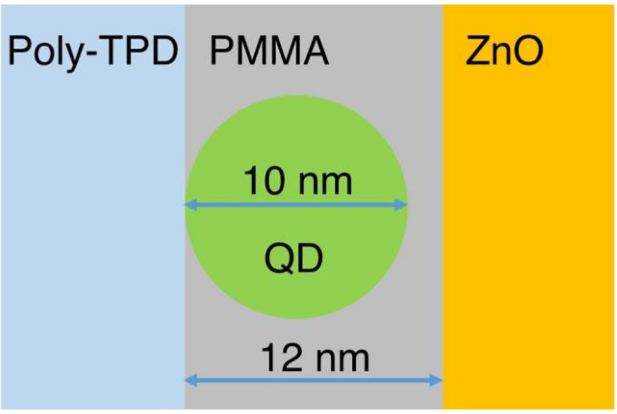

Supplementary Figure 12. Capacitance estimation. Schematic illustration of the structure used to estimate the capacitance of a single quantum dot.

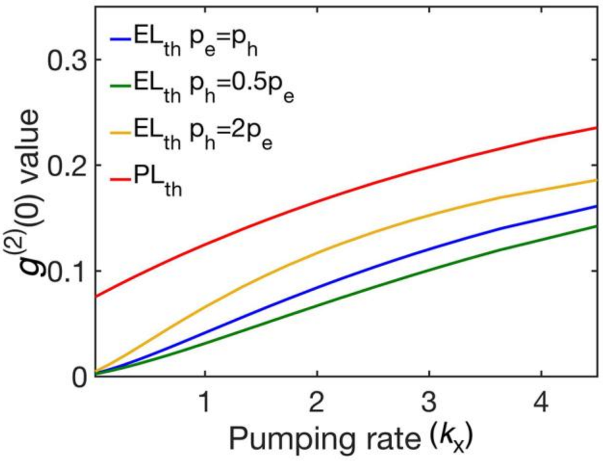

Supplementary Figure 13. The influence of unbalanced charge injection on $g^{(2)}(0)$. The red and blue curves are the same as those plotted in Fig. 4c. Orange and green curves present two conditions which deviate from balanced charge injection, i.e., p_h=0.5p_e and p_h=2p_e respectively. The simulation results reveals that $g^{(2)}(0)$ for single-dot electroluminescence at all conditions are lower than that for the corresponding single-dot photoluminescence (at identical pumping rates).

Supplementary Table

Supplementary Table 1. Fitting parameters used in our model. Measured and calculated ones are presented with different colours.

state	lifetime(ns)	QY	radiative decay time (ns)=lifetime/QY	β-factor[2]	Non-radiative decay time (ns)
X	40	100%	40	1	∞
X^-	3.6	15%	24	1.67	4.23
X^+	0.92	5%	18.4	2.17	0.968
BX	0.76	7.80%	9.74	4.11	0.824
X^{2-}	1.31	6.50%	20	2	1.41
X^{2+}	0.315	2%	13.33	3	0.323

(green)	Measured	(yellow)	From Supplementary Ref. 2	(peach)	Calculated

Supplementary Methods

Carrier dynamics for single-dot electroluminescence

The carrier dynamics can be described by rate equations of a probability distribution of all states in a quantum dot under electrical excitation. Here we only consider states with up to four carriers. This approximation is reasonable because our electroluminescence (EL) experiments were carried out at low-bias conditions. The probability distribution of these states is noted as σ_i, with suffixes i of 1 to 15 representing 1. ground state ($|\mathrm{g}\rangle$), 2. single-hole state ($|\mathrm{h}^+\rangle$), 3. single-electron state ($|\mathrm{e}^-\rangle$), 4. two-hole state ($|2\mathrm{h}^+\rangle$), 5. single-exciton state ($|\mathrm{X}\rangle$), 6. two-electron state ($|2\mathrm{e}^-\rangle$), 7. three-hole state ($|3\mathrm{h}^+\rangle$), 8. positively-charged-exciton state ($|\mathrm{X}^+\rangle$), 9. negatively-charged-exciton state ($|\mathrm{X}^-\rangle$), 10. three-electron state ($|3\mathrm{e}^-\rangle$), 11. four-hole state ($|4\mathrm{h}^+\rangle$), 12. three-hole/one-electron state ($|\mathrm{X}^{2+}\rangle$), 13. bi-exciton state ($|\mathrm{BX}\rangle$), 14. three-electron/one-hole state ($|\mathrm{X}^{2-}\rangle$), and 15. four-electron state ($|4\mathrm{h}^+\rangle$). The rate equations of these states can be expressed as:

$$\frac{d\sigma}{dt} = A \cdot \sigma \tag{1}$$

where $\sigma = [\sigma_1\ \sigma_2\ \sigma_3\ \sigma_4\ \sigma_5\ \sigma_6\ \sigma_7\ \sigma_8\ \sigma_9\ \sigma_{10}\ \sigma_{11}\ \sigma_{12}\ \sigma_{13}\ \sigma_{14}\ \sigma_{15}]^T$ is a column vector consisting of the 15 states in consideration, and A is the coefficient matrix given by:

$$\begin{bmatrix}
-p_e-p_h & 0 & 0 & 0 & k_{\mathrm{X}} & 0 & 0 & 0 & 0 & 0 & 0 & 0 & 0 & 0 & 0 \\
p_h & -\frac{p_e}{\gamma}-p_h\gamma & 0 & 0 & 0 & 0 & 0 & k_{\mathrm{Y}} & 0 & 0 & 0 & 0 & 0 & 0 & 0 \\
p_e & 0 & -p_e\gamma-\frac{p_h}{\gamma} & 0 & 0 & 0 & 0 & 0 & k_{\mathrm{Z}} & 0 & 0 & 0 & 0 & 0 & 0 \\
0 & p_h\gamma & 0 & -\frac{p_e}{\gamma^2}-p_h\gamma^2 & 0 & 0 & 0 & 0 & 0 & 0 & 0 & k_{\mathrm{BY}} & 0 & 0 & 0 \\
0 & \frac{p_e}{\gamma} & \frac{p_h}{\gamma} & 0 & -p_e-p_h-k_{\mathrm{X}} & 0 & 0 & 0 & 0 & 0 & 0 & 0 & k_{\mathrm{BX}} & 0 & 0 \\
0 & 0 & p_e\gamma & 0 & 0 & -p_e\gamma^2-\frac{p_h}{\gamma^2} & 0 & 0 & 0 & 0 & 0 & 0 & 0 & k_{\mathrm{BZ}} & 0 \\
0 & 0 & 0 & p_h\gamma^2 & 0 & 0 & -\frac{p_e}{\gamma^3}-p_h\gamma^3 & 0 & 0 & 0 & \frac{p_e}{\gamma^4} & 0 & 0 & 0 & 0 \\
0 & 0 & 0 & \frac{p_e}{\gamma^2} & p_h & 0 & 0 & -k_{\mathrm{Y}}-\frac{p_e}{\gamma}-p_h\gamma & 0 & 0 & 0 & 0 & 0 & 0 & 0 \\
0 & 0 & 0 & 0 & p_e & \frac{p_h}{\gamma^2} & 0 & 0 & -k_{\mathrm{Z}}-p_e\gamma-\frac{p_h}{\gamma} & 0 & 0 & 0 & 0 & 0 & 0 \\
0 & 0 & 0 & 0 & 0 & p_e\gamma^2 & 0 & 0 & 0 & -p_e\gamma^3-\frac{p_h}{\gamma^3} & 0 & 0 & 0 & 0 & \frac{p_h}{\gamma^4} \\
0 & 0 & 0 & 0 & 0 & 0 & p_h\gamma^3 & 0 & 0 & 0 & -\frac{p_e}{\gamma^4} & 0 & 0 & 0 & 0 \\
0 & 0 & 0 & 0 & 0 & 0 & \frac{p_e}{\gamma^3} & p_h\gamma & 0 & 0 & 0 & -k_{\mathrm{BY}} & 0 & 0 & 0 \\
0 & 0 & 0 & 0 & 0 & 0 & 0 & \frac{p_e}{\gamma} & \frac{p_h}{\gamma} & 0 & 0 & 0 & -k_{\mathrm{BX}} & 0 & 0 \\
0 & 0 & 0 & 0 & 0 & 0 & 0 & 0 & p_e\gamma & \frac{p_h}{\gamma^3} & 0 & 0 & 0 & -k_{\mathrm{BZ}} & 0 \\
0 & 0 & 0 & 0 & 0 & 0 & 0 & 0 & 0 & p_e\gamma^3 & 0 & 0 & 0 & 0 & -\frac{p_h}{\gamma^4}
\end{bmatrix}$$

Here p_e (p_h) stands for the electron-injection (hole-injection) rate of at a certain voltage when the quantum dot is at its neutral state. This charge-injection rate is modified by a coefficient γ

when a net negative (positive) charge exists in the quantum dot due to Coulomb blockade effect. While a positive (negative) charge is presented in the quantum dot, Coulomb attraction is taking into account the electron-injection rate modified by $1/\gamma$ as a first order approximation. The coefficients $k_X, k_Y, k_Z, k_{BX}, k_{BY}, k_{BZ}$ in the matrix denote the decay rates (including radiative and non-radiative decays) of six states as X, X^+, X^-, BX, X^{2+}, and X^{2-}, respectively. Finally, to make the charge circulation a closed loop, we assume that the four-hole (four-electron) state will spontaneously decay to the three-hole (three-electron) states with a rate of p_e (p_h), as we only consider states with up to four carriers. This assumption shall not impact the general conclusion, since the σ_{11} (probability distribution of the four-hole state) and σ_{15} (probability distribution of the four-electron state) are negligibly small in the simulation.

Estimation of γ constant

The modification coefficient γ is estimated by following the model described in Supplementary Ref. 3. We evaluate the energy shift by adding one electron or one hole to a neutral quantum dot using an equation of $E = e^2/C$, where C is the sum of capacitance between the CdSe/CdS quantum dot and two carrier injection layers ($C = C_{QD-ZnO} + C_{QD-TPD}$). Schematic diagram of the quantum dot used in the device is shown in Supplementary Fig. 12. The quantum dot has a core radius of 1.6 nm, a shell thickness of 3.4 nm. Thus the distance from the centre of the quantum dot to the surface of the poly (N,N9-bis(4-butylphenyl)-N,N9-bis(phenyl)-benzidine) (Poly-TPD) layer is ~5 nm, and is ~7 nm to the surface of the ZnO layer with the existence of a ~12-nm thick PMMA layer. The capacitance C is then calculated as 2.33 aF. Therefore, the energy shift is calculated to be 68.6 meV when adding an additional net charge. This result means that a voltage rising of 68.6 mV is required to keep the injection rate constant. Equivalently, from the experimental data shown in Fig. 1f, we get a reduction of EL count rate of ~0.63 times with the decease of applied voltage by 68.6 mV at a driven voltage of ~2.5 V. Therefore, we estimate the modification coefficient γ due to the Coulomb blockade to be 0.63 assuming that the EL count rate has a linear relation with the carrier injection rate.

Numerical simulation of $g^{(2)}(\tau)$

The second order correlation function $g^{(2)}(\tau)$ is proportional to the probability density $J(\tau)$ of detecting a photon at time τ provided that a photon has been detected at time zero. With the rate

equation mentioned in Section 1, we are able to calculate $J(\tau)$ based on the dynamics of six emissive states, i.e., X, X^+, X^-, BX, X^{2+}, and X^{2-}.

$$
\begin{aligned}
g^2(\tau) \propto\, & J(\text{detect one photon at } \tau | \text{detect one photon at } 0) \\
&= \frac{I_X}{I_{total}} J(\text{photon emission at } \tau | \text{X emission at } 0) \\
&+ \frac{I_{X^+}}{I_{total}} J(\text{photon emission at } \tau | \text{X}^+ \text{ emission at } 0) \\
&+ \frac{I_{X^-}}{I_{total}} J(\text{photon emission at } \tau | \text{X}^- \text{ emission at } 0) \\
&+ \frac{I_{BX}}{I_{total}} J(\text{photon emission at } \tau | \text{BX emission at } 0) \\
&+ \frac{I_{X^{2+}}}{I_{total}} J(\text{photon emission at } \tau | \text{X}^{2+} \text{ emission at } 0) \\
&+ \frac{I_{X^{2-}}}{I_{total}} J(\text{photon emission at } \tau | \text{X}^{2-} \text{ emission at } 0)
\end{aligned} \quad (2)
$$

where

$$I_X = \eta_X k_X \sigma_5(\infty) \quad (3)$$

$$I_{X^+} = \eta_{X^+} k_Y \sigma_8(\infty) \quad (4)$$

$$I_{X^-} = \eta_{X^-} k_Z \sigma_9(\infty) \quad (5)$$

$$I_{BX} = \eta_{BX} k_{BX} \sigma_{13}(\infty) \quad (6)$$

$$I_{X^{2+}} = \eta_{X^{2+}} k_{BY} \sigma_{12}(\infty) \quad (7)$$

$$I_{X^{2-}} = \eta_{X^{2-}} k_{BZ} \sigma_{14}(\infty) \quad (8)$$

are the emission intensities from these six states at steady state and I_{total} equals to the sum of six items. The coefficients η_X, η_{X^+}, η_{X^-} η_{BX}, $\eta_{X^{2+}}$ and $\eta_{X^{2-}}$ are quantum yield of X, X^+, X^-, BX, X^{2+}, and X^{2-} states, respectively.

Under weak excitation (p<<min$\{k_X, k_Y, k_Z, k_{BX}, k_{BY}, k_{BZ}\}$), we get:

$$I_{BX} = \frac{\eta_{BX} p^2 \gamma^2}{\bar{k}\beta} + o(p^3) \quad (9)$$

where $\bar{k} = \frac{k_Y k_Z}{k_Y + k_Z}$ and $\beta = \gamma(1 + 2\gamma + 2\gamma^4 + 2\gamma^9 + 2\gamma^{16})$, and

$$I_{total} = \underbrace{\frac{2p\eta_X\gamma}{\beta} + o(p^2)}_{I_X} + \underbrace{\frac{p\eta_{X^+}\gamma^3}{\beta} + o(p^2)}_{I_{X^+}} + \underbrace{\frac{p\eta_{X^-}\gamma^3}{\beta} + o(p^2)}_{I_{X^-}}$$
$$+ \underbrace{\frac{p^2\eta_{BX}\gamma^2}{\bar{k}\beta} + o(p^3)}_{I_{BX}} + \underbrace{\frac{p\eta_{X^{2+}}\gamma^7}{\beta} + o(p^2)}_{I_{X^{2+}}} + \underbrace{\frac{p\eta_{X^{2-}}\gamma^7}{\beta} + o(p^2)}_{I_{X^{2-}}}$$
$$\approx \frac{2p\eta_X\gamma}{\beta} \tag{10}$$

when first order approximation of p and $\eta_{X^+}, \eta_{X^-}\eta_{X^{2+}}, \eta_{X^{2-}} \ll \eta_X$ are taking into account.

The initial conditions following these six emissive events are:

$$\sigma_1 = 1 \text{ (X emission), else equals to 0} \tag{11}$$

$$\sigma_2 = 1 \text{ (X}^+ \text{ emission), else equals to 0} \tag{12}$$

$$\sigma_3 = 1 \text{ (X}^- \text{ emission), else equals to 0} \tag{13}$$

$$\sigma_5 = 1 \text{ (BX emission), else equals to 0} \tag{14}$$

$$\sigma_4 = 1 \text{ (X}^{2+}\text{emission), else equals to 0} \tag{15}$$

$$\sigma_6 = 1 \text{ (X}^{2-}\text{emission), else equals to 0} \tag{16}$$

With these initial conditions, $g^2(\tau)$ can be calculated numerically.

The follow-up state of the six emissive state after photon emission are ground state, $|h^+\rangle, |e^-\rangle$, $|X\rangle$, $|2h^+\rangle$, $|2e^-\rangle$, in which only $|X\rangle$ is still emissive. As a result, only the radiative sequence {BX→X→ground} has contribution to $g^{(2)}(0)$.

So we get:

$$g^{(2)}(0) \propto J(\text{detect one photon at } 0|\text{detect on photon at } 0)$$
$$= \frac{I_{BX}}{I_{total}} J(\text{photon emission at } 0|\text{BX emission at } 0) = \frac{I_{BX}}{I_{total}} k_X\eta_X \tag{17}$$

Dividing by $g^{(2)}(\infty) \propto I_{total}$ for normalization, we get the analytical expression for $g^2(0)$ as:

$$g^{(2)}(0) = \frac{I_{BX}}{I_{total}^2} k_X\eta_X \approx \frac{\frac{\eta_{BX}p^2\gamma^2}{\bar{k}\beta}}{\left(\frac{2p\eta_X\gamma}{\beta}\right)^2} k_X\eta_X = \frac{\eta_{BX}}{\eta_X}\frac{\beta}{4}\frac{k_X}{\bar{k}} \tag{18}$$

Tanking $\gamma = 0.63$ into account, we get $g^{(2)}(0) = 0.41\frac{k_X}{\bar{k}}\frac{\eta_{BX}}{\eta_X}$. Adopting the coefficients listed in Supplementary Table 1, we can estimate $g^2(0)$ of single-dot electroluminescence has a lower limit

which is 0.045 times smaller than the one of single-dot photoluminescence[1] ($g_{PL}^{(2)}(0) = \frac{\eta_{BX}}{\eta_X}$).

Determination of other coefficients

$\boldsymbol{k_X}$**:**

The exciton decay rate is reciprocal to the lifetime of an exciton which was measured from isolated single quantum dots on quartz substrates by the standard TCSPC method. A typical figure is shown in Supplementary Fig. 9d. Lifetime can be acquired by fitting this data with a single exponential decay function. For the decay trace shown in Supplementary Fig. 9d, the exciton lifetime is determined to be 40 ns.

$\boldsymbol{\eta_{\mathrm{X}}}$**:**

For single quantum dot spectroscopy, the single exciton quantum yield is considered as 100% for our non-blinking quantum dots.

$\boldsymbol{k_{\mathrm{BX}}}$**:**

The bi-exciton decay rate is reciprocal to the lifetime of a bi-exciton. Under higher optical pumping power, we can observe the existence of a fast decay channel in single-dot lifetime measurements (Supplementary Fig. 10a). By fitting data with a bi-exponential decay function, we can get the lifetime for the two decay channels. The coefficient of the fast term increases quadratically with the pumping power which is a signature of bi-exciton decay (Supplementary Fig. 10b). Thus, the fast channel with a lifetime of 0.76 ns is attributed to the bi-exciton recombination.

$\boldsymbol{\eta_{\mathrm{BX}}}$**:**

The radiative decay rate of a bi-exciton should be four times faster than that of an exciton[29], namely ~10 ns^{-1} for our dots. Using the equation of $k_{total}=k_r+k_{nr}$, we estimate that k_{nr} for a bi-exciton is ~9 ns^{-1}, which is dominated by Auger process. Finally, $\eta_{\mathrm{BX}} = \frac{k_r}{k_{total}} = 0.076$ is calculated. This value is very close to that from the second-order correlation measurements by pulsed laser excitation (Supplementary Fig. 12, 0.078).

$\mathbf{k_Y}$ **&** $\mathbf{k_Z}$**,** $\boldsymbol{\eta_{X^+}}$ **&** $\boldsymbol{\eta_{X^-}}$**:**

The QY of the positive and negative trion state ($|X^+\rangle, |X^-\rangle$) can be determined from the measured (10 ms time bin, low-power pulsed optical excitation) intensity histograms of single-dot blinking trace (Supplementary Fig. 9). Following the method used in Supplementary Ref. 2, the ‘on’ state

here was attributed to the emission of $|X\rangle$, which has a QY of 100%. Two dim states were attributed to $|X^+\rangle$ and $|X^-\rangle$, respectively. From the intensity ratio between trion state and single exciton state, we got that η_{X^+}=0.05 and η_{X^-}=0.15. Trion emission events were selected according to blinking intensity so that the decay dynamics can be plotted and fitted for $|X^+\rangle$ and $|X^-\rangle$. The lifetimes were determined as 0.92 ns and 3.6 ns for $|X^+\rangle$ and $|X^-\rangle$, respectively.

k_{BY} & k_{BZ}, $\eta_{X^{2+}}$ & $\eta_{X^{2-}}$:

The parameters of $|X^{2+}\rangle$ and $|X^{2-}\rangle$ states cannot be acquired directly from our measurements because these states are rather rare under the optical excitation level. We estimated these parameters according to Supplementary Ref. 2.

Supplementary Table 1 summarizes the measured (green) and the calculated (pink) parameters used in our theoretical model.

Supplementary References:

1. Nair, G., Zhao, J. & Bawendi, M. G. Biexciton quantum yield of single semiconductor nanocrystals from photon statistics. *Nano Lett.* **11**, 1136-1140 (2011).
2. Sampat, S. *et al.*, Multistate Blinking and Scaling of Recombination Rates in Individual Silica-Coated CdSe/CdS Nanocrystals. *ACS Photonics*. **2**, 1505–1512 (2015).
3. Hummon, M. R. *et al.*, Measuring charge trap occupation and energy level in CdSe/ZnS quantum dots using a scanning tunneling microscope. *Phys. Rev. B* **81**, 115439 (2010).
